# Pneumococcal and influenza immunization in asplenic persons: a retrospective population-based cohort study 1990-2002

**DOI:** 10.1186/1471-2334-10-219

**Published:** 2010-07-22

**Authors:** Joanne M Langley, Linda Dodds, Deshayne Fell, G Ross Langley

**Affiliations:** 1 Department of Pediatrics, Dalhousie University, IWK Health Centre, 5850 University Avenue, Halifax Nova Scotia, B3K 6R8, Canada; 2 Department of Community Health & Epidemiology, Dalhousie University, Centre for Clinical Research, 5790 University Avenue, Halifax, NS B3H 1V7, Canada; 3 Canadian Center for Vaccinology, IWK Health Centre and Dalhousie University, 5850 University Avenue, Halifax Nova Scotia, B3K 6R8, Canada; 4 Department of Obstetrics and Gynecology, IWK Health Centre and Dalhousie University, 5850 University Avenue, Halifax Nova Scotia, B3K 6R8, Canada; 5 Department of Medicine, Dalhousie University, QEII Health Sciences Centre, Halifax, NS, B3H 2Y9, Canada

## Abstract

**Background:**

Splenectomy is associated with increased risk for bacteremia, due to impaired clearance of bloodborne agents and to altered phagocytosis and humoral immunity. We conducted a retrospective cohort study of patients at risk for splenectomy for a 13-year period to determine immunization coverage, and outcomes of those with and without splenectomy, and with or without receipt of influenza or pneumococcal vaccine.

**Methods:**

Data were extracted from the provincial Medical Services Insurance database for insured services rendered by a physician for 1990-2002, and from the Vital Statistics Death database. The eligible cohort was selected based on diagnostic codes for hematologic conditions for which splenectomy might be considered, such as immune thrombocytopenia. Each patient was followed longitudinally from the date of first diagnosis until 31Dec2002, or death, or relocation out-of province. In addition, persons with splenectomy and no hematologic condition were identified and followed for 6 months post-surgery. Infectious illness rates per 100 person-years of observation and death rates were calculated with and without splenectomy. Death rates were determined using splenectomy status as a time-dependent covariate. The relationship between splenectomy and death according to immunization status was examined using Cox proportional hazard ratios.

**Results:**

Of 38,812 persons in the cohort 427 subjects with a hematologic diagnosis had splenectomy and another 452 subjects without a hematologic diagnosis had this surgery. 72% were > 18 years of age. Pneumococcal immunization was recorded in 16.5% of asplenic patients overall, and was not associated with reduced risk of death in these persons (adjusted Hazard Ratio [HR] = 1.07, 95% CI 0.70 - 1.65). Influenza immunization was recorded in 53.1% of asplenic patients overall, and was associated with reduced risk of death (adjusted HR = 0.46, 0.33-0.62). No pneumococcal or influenza immunization was recorded in patients with a hematologic diagnosis without splenectomy. Infectious illness visits were higher among all patients who had a splenectomy than among those without a splenectomy (151 visits/100 person-years of observation in the post-splenectomy period vs. 120 visits/100 person-years; p < 0.0001).

**Conclusions:**

In asplenic patients, influenza immunization is associated with a 54% reduced risk of death compared to unimmunized asplenic persons; no reduction in risk was demonstrated with (polysaccharide) pneumococcal vaccine. Vaccine coverage in the entire cohort was less than routinely recommended. Improved delivery of infection prevention programs to this population is warranted. Conjugate pneumococcal vaccines should be urgently studied in this immunocompromised population.

## Background

Splenectomy is associated with increased risk for bacteremia due to impaired clearance of bloodborne agents, and to altered phagocytosis and humoral immunity[1-4]. The spleen is also the main site of immunoglobulin M synthesis, and low levels of the opsonins tuftsin and properdin have been reported post-splenectomy [5]. In a review of 78 studies with 19 680 asplenic patients, 3.2% developed invasive infection and the overall mortality was 1.4% [6]. For those who develop fulminant, overwhelming post-splenectomy sepsis, mortality rates approach 50% [7], and those who survive may be left with significant morbidity. *Streptococcus pneumoniae *is the most common etiology of serious infection in this population[8], but illness due to other encapsulated organisms such as *Haemophilus influenzae *and *Neisseria meningitidis*, occurs.

Although infection prevention strategies such as immunization, antibiotic prophylaxis and early care for febrile illness have been recommended for asplenic patients for decades, implementation is often not optimal [8-15]. Life-threatening infectious complications of splenectomy continue to occur. Accordingly, we reviewed outcomes of a province-wide cohort of patients who had health conditions for which splenectomy might be considered or indicated over a 13-year period to determine vaccine coverage rates, if patients with splenectomy have different infection and mortality rates than patients without splenectomy, and whether immunization was associated with better outcomes.

## Methods

### Data sources

A population-based retrospective cohort was assembled by linking several comprehensive provincial health databases. Nova Scotia is a province in Eastern Canada with a population of ~ 900,000. All residents have a unique health card number which is used to track health services in the universal health care system, to reimburse providers and to track persons moving in and out of the province. The cohort consisted of all persons resident in the province from 1990-2002, who had health conditions for which splenectomy might be considered or indicated.

Data were extracted from the Nova Scotia Medical Services Insurance (MSI) database for insured services rendered by a physician for 1990-2002 and from the provincial Vital Statistics Death Database. The MSI database is a single comprehensive administrative database for health services. All physicians and all residents in the province for more than three months were participants in the health insurance plan. Recording and reporting of services given was the only method of physician remuneration in this largely fee-for-service system and regular ongoing auditing confirms services are given. The cohort of persons at risk for splenectomy was selected by identifying all International Classification of Diagnostic Codes - 9^th ^Clinical Modification 9 (ICD-CM9) for health conditions for which splenectomy might be considered or indicated. These codes were grouped into five categories, and all patients with a first field diagnostic code for at least one of the following categories were included: immune thrombocytopenia (ITP), Hodgkin's disease, non-Hodgkin lymphoma, haemolytic anemia, or hypersplenism.

Other variables extracted included the following: birth date, diagnosis and service(s), immunizations delivered, whether a splenectomy was performed and the date of surgery, physician visits for which an infectious disease code was used ("infectious illness visit"), and, if applicable, moving out of the province or death. Antibiotic use was not recorded in these datasets. Each patient was followed from the date of first diagnosis until 31 Dec 2002, or death, or relocation out-of province. In addition, an extraction was completed for patients who had a splenectomy during the same time period but did not have one of the aforementioned health conditions. These asplenic persons were assumed to have had a splenectomy on a non-elective basis. The codes for these concurrent events were diverse and included trauma and other surgeries. An arbitrary time period of 6 months was selected to follow health outcomes post-splenectomy.

The conjugate heptavalent pneumococcal vaccine Prevnar^® ^was brought into routine immunization programs between 2001 and 2003 in Canada and therefore it is assumed that the 23-valent pneumococcal polysaccharide vaccine was the only pneumococcal vaccine used to immunize the population in this study. The influenza vaccine used in Canada is a trivalent inactivated injectable formulation.

### Statistical analysis

The outcome measures of interest were the number of patients living with asplenia (in order to plan health care program delivery), the immunization history of asplenic persons compared to those without splenectomy, and the incidence of infectious illness visits and death in these high risk persons according to immunization status and splenectomy.

Categorical and continuous variables were analyzed using descriptive statistics. Median, 25^th ^and 75^th ^percentiles were used where variables were not normally distributed. In the case of cell counts less than five, the numbers are not reported.

Infectious illness rates were expressed per 100 person-years of observation in persons with splenectomy and without splenectomy. Rates of death were calculated per 1,000 person years of observation, by splenectomy status and by immunization status. The relationship between splenectomy and death was assessed using Cox proportional hazards regression models (adjusted and unadjusted) using a time-dependent covariate approach whereby the follow-up time for subjects with splenectomy is divided into pre-and post-splenectomy. The relationship between any pneumococcal or influenza immunization and death was evaluated using standard survival analysis among the subset of subjects who had a splenectomy. Survival curves were adjusted for age at diagnosis, gender and diagnostic category. The "end date" for the study was assigned using the termination date if the subject moved or died, or the end of the study period (31 December 2002).

All analyses were performed using SAS (SAS Institute Inc., Cary NC).

The study protocol was approved by the Research Ethics Boards of the IWK Health Centre and the Capital Health District, Halifax, Nova Scotia and by the Privacy Officer of the Nova Scotia Department of Health.

## Results

### Demographics

The total cohort of patients with hematologic diagnoses of interest and with splenectomy but no pre-existing hematologic diagnosis consisted of 38,812 patients; 72% were >18 years of age (n = 28,096). Females comprised 57% of the cohort (22,095/38,812). The most common hematologic diagnosis was hypersplenism (78% of children, 44% of adults) followed by ITP (11% of children, 23% of adults). Splenectomy occurred in 427 patients with a hematologic diagnosis in the cohort (1.2%), and in another 452 subjects without a hematologic diagnosis (431 adults; 21 children). Thus over the 13 years, 879 patients, or about 68 persons each year, had splenectomy. Characteristics of the asplenic patients are seen in Table 1.

**Table 1:** Characteristics of 879 asplenic patients

Characteristic	Total		Pre-existing hematologic diagnosis for which splenectomy could be considered										No pre-existing hematologic diagnosis	
			ITP		Hodgkins disease		Non-Hodgkins lymphoma		Hemolytic anemia		Hypersplenism			
	n = 879		n = 196		n = 9		n = 89		n = 55		n = 78		n = 452	
	(100%)		(22.3%)		(1.0%)		(10.1%)		(6.3%)		(8.9%)		(51.4%)	
	n	%	n	%	n	%	n	%	n	%	n	%	n	%
Age at splenectomy														
≤ 18 years	65	7.4	12	6.1	†		†		19	34.6	11	14.1	21	4.7
> 18 years	814	92.6	184	93.9					36	65.4	67	85.9	431	95.3
Gender														
Male	471	53.6	79	40.3	†		54	60.7	27	49.1	41	52.6	265	58.6
Female	408	46.4	117	56.7			35	39.3	28	50.9	37	47.4	187	41.4
Death														
Yes	230	26.2	39	19.9	†		28	31.5	13	23.6	12	15.4	135	29.9
No	649	73.8	157	80.1			61	68.5	42	76.4	66	84.6	317	70.1
Any vaccination for influenza														
Yes	467	53.1	123	62.8	†		49	55.1	39	70.9	41	52.6	210	46.5
No	412	46.9	73	37.2			40	44.9	16	29.1	37	47.4	242	53.5
Any vaccination for *S.pneumoniae*														
Yes	145	16.5	42	21.4	†		12	13.5	16	29.1	21	26.9	53	11.7
No	734	83.5	154	78.6			77	86.5	39	70.9	57	73.1	399	88.3

ITP = immune thrombocytopenic purpura

† Numbers suppressed due to cell size < 5

Splenectomy was most commonly performed for ITP (4.04 splenectomies/1000 person years). Rates for other hematologic conditions were hemolytic anemia (3.63/1000 person years), Non-Hodgkins lymphoma (2.84/1000 person-years), Hodgkin's disease (1.32/1000 person years) and hypersplenism (0.40/1000 person-years).

### Vaccine coverage

**Figure 1:**
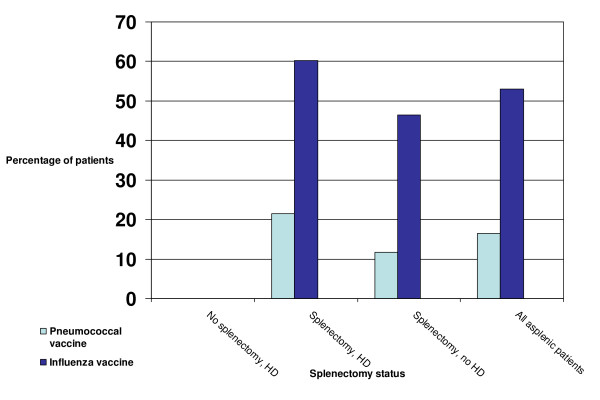
**Percentage of patients with immunization according to splenectomy status, Province of NS 1990-2002**. HD = Hematologic disorder.

Immunization against *Streptococcus pneumoniae *was recorded in 16.5% (n = 132) of all asplenic patients, but not recorded in any patients with a hematologic diagnosis without splenectomy (Figure 1). Influenza immunization was recorded in 53.1% (467/879) of asplenic patients, and 140 patients had at least one influenza vaccination, 79 had two, 62 had 3, 52 had 4, 49 had 5, 48 had 6, 24 had 7, 7 had 8, and 9 had 4 influenza vaccines. The median number of patients receiving both vaccines was 137. Both forms of immunization were recorded more frequently among asplenic patients with a pre-existing hematologic condition than in those without a pre-existing hematologic condition.

### Outcomes

Infectious illness visits (IIV) were higher among all patients who had a splenectomy than among those without a splenectomy (151 visits/100 person-years of observation in the post-splenectomy period vs. 120 visits/100 person-years; p < 0.0001). Among subjects who had a splenectomy, IIV were decreased in the period post-splenectomy compared to the period before splenectomy for all subgroups except among those patients with ITP. In the period before surgery, overall the rate of IIV was 265 per 100 person years of observation, whereas post-surgery the rate of IIV was 151 per 100 person years of observation.

Among subjects with a hematologic condition, results from Cox proportional hazards regression models, in which splenectomy was modeled as a time-dependent covariate, indicated that the risk of death was significantly higher for patients who underwent splenectomy compared with those who did not (Table 2).

**Table 2:** Hazards ratios (HR) and 95% confidence intervals (CI) for the relationship between splenectomy (Vs. no splenectomy) and death among subjects with a hematologic condition*

Diagnosis category	Death rates (per 1000 person-years)		**HR, 95% CI**^**†**^
	Splenectomy	No splenectomy	
ITP	36.28	26.21	1.57, 1.08-2.29
Hodgkins disease	57.98	26.61	6.47, 2.83-14.81
Non-Hodgkins lymphoma	75.30	56.29	1.58, 1.05-2.39
Hemolytic anemia	45.18	21.80	3.40, 1.76-6.57
Hypersplenism	19.68	5.05	1.16, 0.59-2.26
*Overall (all patients with a hematologic condition) *‡	39.65	15.34	1.75, 1.37-2.24

ITP = immune thrombocytopenic purpura

* Rates and HR calculated using the time-dependent covariate approach where follow-up time for subjects with splenectomy is divided into pre- and post-splenectomy

† Adjusted for age at diagnosis and gender.

‡ Adjusted for age at diagnosis, gender and diagnostic category.

In subgroup analysis the HR for the association between splenectomy and death was not different for conditions which were considered malignant (Hodgkins disease, non-Hodgkins lymphoma) compared to those considered non-malignant conditions (ITP, hemolytic anemia, hypersplenism): was 1.74 (1.22-2.48). v. 1.75 (1.36-2.54).

Among asplenic subjects, pneumococcal immunization, adjusted for age at diagnosis, gender and diagnostic category, was associated with a reduced risk of death when the effect of influenza vaccine was not considered (Hazard Ratio [HR] 0.68, 95% CI 0.47-1.00). However, no protective effect of pneumococcal vaccine was observed when the analysis was adjusted for the effect of influenza vaccination: HR 1.07 (95% CI 0.70-1.65).

The risk of death was reduced among those aplenic persons who had at least one influenza vaccine: HR (adjusted for pneumococcal vaccine) 0.46 (95% CI 0.33-0.62). In subgroup analysis this effect was seen in ITP, hemolytic anemia, and splenectomy without a pre-existing hematologic diagnosis, but not in the other groups (Table 3).

**Table 3:** Deaths rates and hazard ratios (HR) with 95% confidence intervals (CI) for the independent relationship between pneumococcal vaccination (vs. no pneumococcal vaccination), and influenza vaccination (vs. no influenza vaccination) and death among asplenic subjects

Diagnosis category	Pneumococcal vaccination			Influenza vaccination		
	Death rates (per 1000 person-years)		HR, 95% CI*	Death rates (per 1000 person-years)		HR, 95% CI*
	Pneumococcal vaccination	No pneumococcal vaccination		Influenza vaccination	No influenza vaccination	
ITP	16.80	32.35	0.91, 0.33-2.54	16.00	52.79	0.36, 0.18-0.74
Hodgkins disease	0.00	53.50	Indeterminate	23.97	74.21	indeterminate
Non-Hodgkins lymphoma	45.77	59.02	1.00, 0.32-3.09	42.59	75.37	0.45, 0.19-1.08
Hemolytic anemia	25.10	38.78	1.89, 0.31-11.44	17.65	84.98	0.10, 0.12-0.55
Hypersplenism	15.77	18.16	3.21, 0.32-32.13	10.16	27.37	0.17, 0.02-1.38
Splenectomy, no pre-existing hematologic diagnosis	41.81	57.21	1.11, 0.61-2.01	39.58	68.43	0.52, 0.34-0.78
*Overall (all asplenic patients) *^†^	28.44	45.78	1.07, 0.70-1.65	27.00	61.67	0.46, 0.33-0.62

ITP = immune thrombocytopenic purpura

* Adjusted for age at diagnosis and gender.

† Adjusted for age at diagnosis, gender and diagnostic category.

The hazard ratio for the association between influenza immunization and death for the non-malignant group is 0.35 (0.21-0.58) and for the malignant subgroup is 0.48 (0.22-1.04). For pneumococcal immunization these HRs are 0.32 (0.17-0.58) in the non-malignant subgroup and 0.49 (0.22-1.10) for the malignant group.

**Figure 2:**
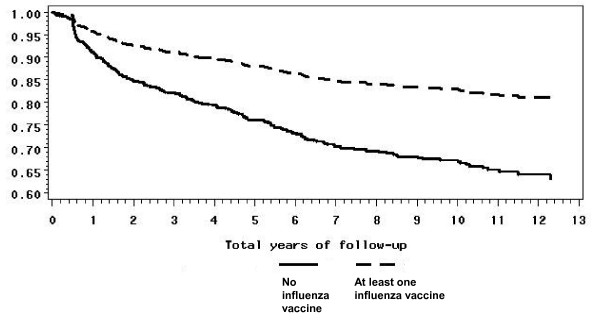
**Age and sex-adjusted survival curves for persons with a splenectomy and any hematologic condition, with and without influenza vaccination**. Y axis = Probability of surviving, X axis = years after splenectomy

Figure 2 presents the survival curve of the relationship between death and influenza immunization in immunized and unimmunized persons with splenectomy and any hematologic condition.

## Discussion

This study shows a 53% reduced risk of death in asplenic persons who received influenza immunization compared to those who had no record of such immunization; but surprisingly no influence on death was seen for pneumococcal immunization. Overwhelming post-splenectomy infection (OPSI), usually due to *Streptococcus pneumoniae*, is perhaps the best known infectious complication of asplenia and thus pneumococcal immunization is universally recommended. Immunization against other encapsulated organisms such as *H. influenzae *and *Neisseria meningitidis *is also recommended, preferably at least two weeks prior to splenectomy. Annual influenza vaccine, as well as age appropriate immunization, is also considered standard of care.

Receipt of influenza vaccine was associated with reduced risk of death in the whole cohort and in three subgroups (asplenic patients without a hematologic condition, with ITP and with hemolytic anemia). The mechanism of this protection is not clear, and no randomized controlled trials of influenza vaccine efficacy in asplenic persons have been performed. Asplenic persons are not generally thought to be at increased risk for complicated viral infections, since innate and cell-mediated immunity are intact. However, production of antibody involves interaction between the humoral and cell-mediated immune systems which could be altered in asplenic persons, and cases of serious viral infection in asplenic persons have been reported[16]. The association between influenza infection and secondary bacterial pneumonia is established; this may be the mechanism by which influenza vaccine protects asplenic persons. This protection, if real, is not well studied in this immunocompromised population. Indeed, a Medline search using the MESH terms "influenza vaccines" AND "splenectomy" or "spleen (subheading abnormalities)" back to 1966 revealed many guidelines, but only one human study of influenza vaccine in persons with splenectomy[17], one in persons with Hodgkin's disease[18] and a letter to the editor[19]. In 62 patients with splenectomy compared to 55 healthy controls, serologic responses met regulatory criteria regardless of the time from splenectomy, with seroprotection outcomes ranging from 62.9 to 90.3%. Our finding of the protective effect of influenza vaccine could be important for improved health for asplenic and hyposplenic persons, and should be confirmed in other studies.

Immunization coverage overall was very low for (16.5% for *S. pneumoniae) *but better for influenza (53.1%). It is unlikely that our finding of poor immunization coverage is restricted to our jurisdiction, as others have noted incomplete physician and patient knowledge of risks and preventive interventions, poor vaccine coverage, and a lack of systematic immunization programs[9-15,20]. If influenza vaccine offers the protection observed in this study, the need for improved delivery of vaccine will be necessary.

There is concern that asplenic persons may demonstrate reduced immunogenicity to vaccines. In a few small studies variable immunogenicity has been demonstrated for polysaccharide vaccines [21-23], but better immune responses were seen for protein conjugate vaccines [24-28]. The pneumococcal polysaccharide vaccine was in use during the time period of the study. The ongoing introduction of highly effective and immunogenic protein conjugate vaccines for *H. influenzae, S. pneumoniae *and *N. meningitides *has the potential to offer asplenic persons both direct and indirect protection from life-threatening infection. Indirect protection of unimmunized older persons against invasive pneumococcal disease has been shown following the introduction of universal infant immunization with heptavalent protein conjugate *S. pneumoniae *vaccine [29,30]. Little effort has been directed towards investigation of how these vaccines could be best used in asplenic person in order to determine optimum schedules, doses and timing of immunization, and we suggest that it is urgently needed.

There are several limitations to the design of this study. Because health-care utilization data was collected if a patient sought care, patients with health-seeking behaviour may be more likely to be identified with this method than in a prospective study where active surveillance could be performed. However, it should be noted that this would be unlikely to preferentially favor influenza over pneumococcal immunization. As well one of our outcomes was death as determined by a governmental Vital Statistics database, which would not have been subject to any biases. Comprehensive risk factor information is not collected in these datasets and so there may be confounders for splenectomy, physician visits, death and for immunization which could contribute to outcomes but are not controlled for in the analysis. For example, prophylactic antibiotic use, recommended by many authorities for up to five years to life after splenectomy, may be more common in patients who receive immunization from their care providers. Of note, we did control for age at diagnosis, sex, hematologic condition, time since diagnosis and splenectomy status. A further limitation is short follow-up for after splenectomy for persons without hematologic conditions; future studies should ensure follow-up of several years. Finally, this population likely underestimates the burden of infection in asplenic persons, since those with functional asplenia, hyposplenia or congenital asplenia would not have been identified.

In addition to immunization, other preventive interventions are recommended to avoid devastating and life-threatening complications of infection in persons with splenectomy [3,31]. These include early medical attention and antibiotic therapy for infectious illness, antibiotic prophylaxis, patient education about risks associated with infection, travel and animal bites, and wearing an alert bracelet, pendant or card, so that health professionals will be aware of their status. The literature is replete with surveys showing that recommendations for the prevention of infection are often not being followed by health care providers and patients, and that many asplenic patients are not aware of the risk associated with their acquired immunodeficiency [8,12,20,32,11,15]. An ideal system to ensure compliance with guidelines has not been reported, but a post-splenectomy registry may provide a systematic approach to this population, and could be cost-effective in terms of avoidance of mortality and overwhelming post-splenectomy sepsis [33]. Based on the results of this study, we plan to develop a method for contacting asplenic persons in our province to determine their health education needs, and their willingness to join a registry so that appropriate immunization and other interventions can be offered over time.

## Conclusions

In a retrospective population-based cohort study from 1990-2002 of persons with splenectomy, influenza immunization was associated with a 54% reduced risk of death compared to unimmunized asplenic persons. No reduction in risk of death was demonstrated with pneumococcal vaccine. Vaccine coverage for both pneumococcal and influenza vaccines in the entire cohort was less than routinely recommended (16.5% and 53.1% respectively). Improved delivery of infection prevention programs to this population is warranted. Conjugate pneumococcal vaccines should be urgently studied in this immunocompromised population.

## Competing interests

In the past five years none of the authors have received reimbursements, fees, funding, or salary from an organization that may in any way gain or lose financially from the publication of this manuscript, either now or in the future. None of the authors hold any stocks or shares in an organization that may in any way gain or lose financially from the publication of this manuscript, either now or in the future. There are no relevant patents to declare. None of the authors have any other financial competing interests.

### Non-financial competing interests

There are no non-financial competing interests (political, personal, religious, ideological, academic, intellectual, commercial or any other) to declare in relation to this manuscript.

## Authors' contributions

GRL conceived the study and obtained the funding. GRL and JML designed the study. LD and DF designed and performed the statistical analysis. JML prepared the manuscript; and all authors contributed to subsequent revisions and approved the final version.

## Pre-publication history

The pre-publication history for this paper can be accessed here:

http://www.biomedcentral.com/1471-2334/10/219/prepub
